# Deciphering temporal scales of visual awareness: insights from flicker frequency modulation in continuous flash suppression

**DOI:** 10.1093/nc/niaf005

**Published:** 2025-03-06

**Authors:** Ishan Singhal, Narayanan Srinivasan

**Affiliations:** Department of Cognitive Science, Indian Institute of Technology Kanpur, Kanpur, Uttar Pradesh 208016, India; Centre for Developing Intelligent Systems, Indian Institute of Technology Kanpur, Kanpur, Uttar Pradesh 208016, India; Department of Cognitive Science, Indian Institute of Technology Kanpur, Kanpur, Uttar Pradesh 208016, India

**Keywords:** hierarchy, multiple timescales, visual awareness, time consciousness, continuous flash suppression

## Abstract

Evidence from temporal regularities in perception, temporal phenomenology, and neural dynamics indicate that our awareness evolves and devolves over several timescales. However, most theories of consciousness posit a single timescale of processing at the end of which a percept is rendered conscious. To show evidence for multiple timescales, we utilized continuous flash suppression (CFS). Based on a hierarchical framework of temporal phenomenology, we reasoned that different flicker rates (1, 4, 10, and 25 Hz) of the suppressor should be able to perturb phenomenologically distinct tasks. We designed four experiments that used different perceptual tasks (*N* = 48). The results showed that entry of contents into conscious awareness, their attentional sampling, perceptual grouping, and exiting from awareness were all maximally perturbed at distinct flicker frequencies of the suppressor in a CFS paradigm. Our demonstration shows that different flicker frequencies perturb different phenomenological aspects of awareness, and these flicker frequencies systematically map onto temporal hierarchies of timing of awareness.

## Introduction

How do contents of our visual perception unfold over time? Are there temporal regularities to how visual content enters and exits awareness? For the last three decades and more, evidence supporting the thesis that awareness evolves in parallel over multiple timescales has been growing. This has come from clustering the timing of perceptual processes over distinct timescales (Pöppel 1997, 2004), temporal phenomenology (Singhal and Srinivasan 2021), time perception (Wittmann and Van Wassenhove 2009), and neural dynamics involved in awareness (Varela 1999, Jirsa and Müller 2013, Fries 2015). Consider for instance the timescales of different kinds of masking, which prevent awareness of briefly presented targets when a mask is presented before or after the target. In these studies, the duration at which the masking effect is maximum (30–80 ms) and the temporal extent of masking effects (∼300 ms) are different [see Breitmeyer and Ogmen (2006) for a review]. Similarly, in binocular rivalry, dynamics of two timescales are at play: the dwell time of each percept (3–5 s) and the time gap at which two disparately presented stimuli still show binocular rivalry (∼300 ms; van Boxtel et al. 2008).

Hierarchical models of temporal evolution of awareness attribute these results to distinct intrinsic timescales of perceptual processing in the brain (Golesorkhi et al. 2021) and different phenomenological levels of experienced content (Singhal and Srinivasan 2021). Neuro-physiologically, oscillatory processes across different frequency bands are thought to index the distinct temporal regularities of different perceptual processes (binding, integration, identification, attentional sampling, etc.) involved in awareness (Jirsa and Müller 2013, Fries 2015, Golesorkhi et al. 2021). Distinct timescales of awareness are also proposed to be linked to the different latencies of the two visual pathways (parvocellular and magnocellular), for instance, in explaining visibility under different masking and priming paradigms (Breitmeyer and Ogmen 2006) and in the experience of temporal illusions (Piper 2019). Here, oscillatory processes at different frequencies are thought to interact via phase and amplitude coupling to constrain contents of awareness (Jirsa and Müller 2013, Piper 2019, Singhal and Srinivasan 2021).

However, these findings have not made headway in changing the way consciousness theories formulate evolution and devolution of visual content. Almost all theories of consciousness posit single timescales of processing (in the range of 100–500 ms) at the end of which a percept is rendered conscious (Kent and Wittmann 2021). The only disagreement between the theories of consciousness regarding time is how long it takes for people to become aware of a novel stimulus (Förster et al. 2020). We developed a new variant of the continuous flash suppression paradigm to allow findings and evidence from temporal phenomenology to inform theories of consciousness. Continuous flash suppression (CFS) is a paradigm based on binocular rivalry, where a flickering suppressor temporarily prevents awareness of a visual target (Tsuchiya and Koch 2004, 2005). We reasoned that in modifying the flicker frequencies in this established paradigm used to study visual awareness, we could systematically demonstrate the evolution and devolution of awareness over multiple timescales.

### Present study

**Figure 1:**
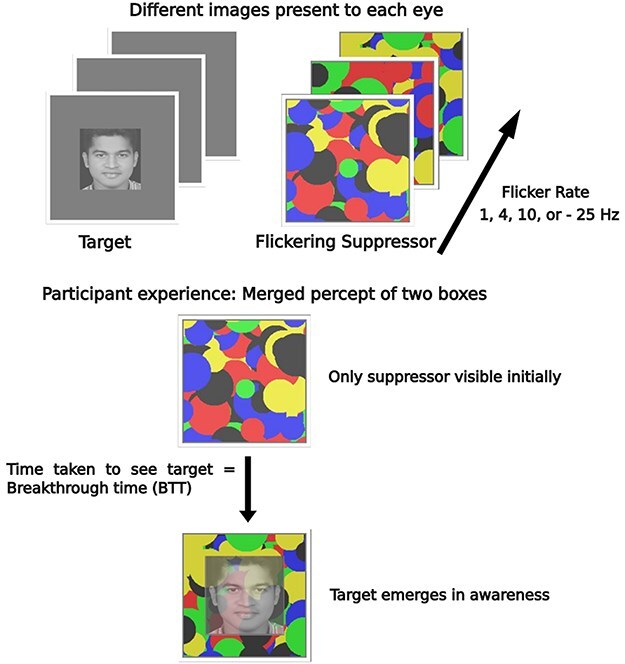
Illustration of a CFS paradigm. A target and a suppressor are presented to individual eyes. The participants see a merged percept, and slowly the target becomes visible. Breakthrough times are measured, as a function of tasks and flicker rates.

The logic behind our paradigm comes from double-dissociation studies of perturbations and lesions in neuroscience. Our study employed this same logic inside the CFS paradigm (Fig. 1), except to see perturbations in time. We selected four flicker frequencies (1, 4, 10, and 25 Hz) of the suppressor inside a CFS task to show that they uniquely perturb distinct aspects of visual awareness in four separate tasks. This was based on the assumption that different flicker frequencies of the suppressor would perturb distinct oscillatory processes of the perceptual system. In our previous work, we detailed the phenomenological nature of these different oscillatory processes (Singhal and Srinivasan 2021). Here, we set out to show that the flicker rate of the suppressor would impair performance in a manner that was phenomenologically distinct. For example, when the suppressor flickers at 4 Hz, it would maximally and selectively impair one aspect of visual awareness, and not others (similarly for other flicker frequencies). Our aim was to show that (i) different flicker frequencies maximally hinder different kinds of perceptual tasks or contents, (ii) these flicker frequencies systematically map onto existing literature on timing of cognition, and, correspondingly, (iii) the phenomenological nature of these different perceptual tasks also maps onto unique phenomenological modes within existing nested hierarchical models of time-consciousness.

The rationale for choosing these tasks comes from previous work on developing a hierarchical multi-timescale framework for combining models of temporal phenomenology (Singhal and Srinivasan 2021). This previous work proposed that any given moment of experience has at least three distinct phenomenological modes over which visual content evolves. The cinematic aspect of our experience (which updates too fast to have a temporal extent in our experience) updates over timescales of 30–50 ms, the tail of the comet of experience, which has perceptual echoes of just-past contents that fade over 3–5 s (retentional aspects of experience), and finally, the content of our experience that has a phenomenological temporal breadth and extent updates over 200–500 ms. To gauge these different phenomenological timescales, we choose flicker frequencies of 25, 1, and 4 Hz (corresponding to cinematic, retentional, and extensional models). Next, we chose tasks that aligned with these timescales and phenomenological modes.

**Figure 2:**
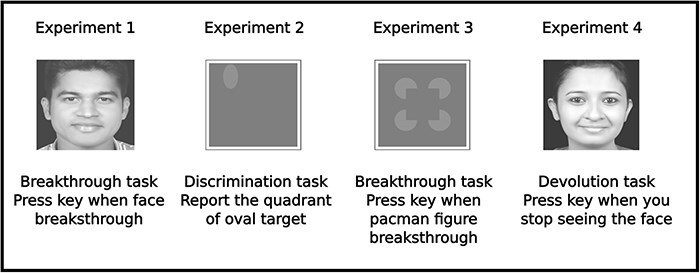
Experiments 1–4, including a brief description of their tasks, along with sample stimuli.

To investigate the regularity at which visual contents break into awareness, we used a breaking suppression version of CFS (bCFS). Here, participants are simply asked to report as soon as they see a target image [see Pournaghdali and Schwartz (2020) for review]. We hypothesized that this task would be maximally inhibited when the suppressor flickered at 4 Hz, given prior evidence of contents breaking into awareness in temporal cycles of ∼250 ms (Singhal and Srinivasan 2021). Similarly, to demonstrate that early visual Gestalt-like grouping of visual content occurs at a faster timescale, we asked participants to report breakthroughs of subjective contours that can be seen using pacman figures. These figures were either grouped to form an illusory square or not (Fig. 2). Studies have shown an advantage for illusory contours to break faster into awareness (Wang et al. 2012). We hypothesized a null effect, specifically that this advantage would disappear at the fastest flickering frequency (25 Hz) since the perceptual mechanisms responsible for this priority would be perturbed. Along the same lines, we created another novel variant of the CFS task to investigate the regularity at which visual contents exit our awareness (reciprocal CFS). We first presented target images to participants at maximum contrast and then slowly ramped down the contrast of the image when the suppressor flickered at different rates. We hypothesized that the image would slowly disappear from participants’ awareness when the flicker frequency matched the retentional timescales of awareness (∼1 Hz). This was because we expected a slow flicker frequency to perturb the process that retains perceptual echoes of just-past events, and prior literature has argued that these processes operate over a few seconds (Dainton 2010, Kent and Wittmann 2021, Singhal and Srinivasan 2021).

To benchmark these modifications against the standard use of CFS, we also used a flicker rate of 10 Hz. The reason this flicker frequency was originally used was on the hypothesis of a single timescale of consciousness at the end of which contents enter awareness (cycles of 100 ms at ∼10 Hz; Tsuchiya and Koch 2005). It is possible that this flicker frequency was chosen in the original design because it matches the putative range of rhythmic attentional sampling (Zhu et al. 2016). Thus, we also used a spatial attention task specifically to see if it was maximally inhibited at 10 Hz.

### Choice of stimuli

We chose stimuli that were relevant for the four tasks of CFS. For the tasks where we were interested in contents entering and exiting from visual awareness (Experiments 1 and 4), we used images of faces. This is because previous work has shown face stimuli are prioritized for breakthrough under CFS [see Stein (2019) for a review]. Thus, by using images of faces we ensured that temporal regularities of breaking suppression and stimuli getting suppressed were revealed through a stimulus category that has been shown to work well with CFS paradigms. For the task where we wanted to highlight the role of attention (Experiment 2), we chose to present an oval-shaped object, which was hidden in one of the quadrants inside the display. We did this to ensure that participants needed to spatially sample the display to locate the target and to increase target-suppressor similarity (oval target and circular objects in the suppressor, respectively). These were done under the assumption that both spatial attention and feature-based attention would be engaged in doing this task. Similarly, to study the temporal regularity of early-visual grouping (Experiment 3), we used a Kanizsa-square configuration made up for pacmen-like circles. Here too, we used a breaking suppression task, allowing for both face and nonface stimuli to be used for a similar task.

## Experiment 1

### Materials and Methods

#### Participants

In each experiment of this study, we ran 12 participants (mean age = 25.6 years; three females). This was based on conservative estimates of effect sizes from similar previous studies (Han et al. 2016). On halving the effect sizes from this study (*η_p_* = ∼0.3), we derived a required sample size of 12 to obtain reasonable power (0.8).

#### Stimuli and apparatus

The frames for the flickering suppressors for all four experiments were generated from the CFS Crafter library in MATLAB (Wang et al. 2023). The crafter tool was developed to ensure control over flicker frequencies of the suppressor in CFS paradigms, given that previous results have shown that the lack of this control leads to low frequencies dominating the suppressor (Han et al. 2018). Here, we used the tool to ensure that the four flicker frequencies of interest were indeed maximally dominant in each individual suppressor series when generating the masks. The suppressor images were drawn from a large pool of generated masks (100) without repetition in subsequent frames or within a period of a particular flicker frequency. The target images were pictures of male and female faces. Pictures of two male and female faces were drawn from a pre-existing database (Grewal et al. 2012). These pictures were already comparable on mean luminance and valence ratings.

All experiments in this study used the same apparatus. Participants were seated ∼125 cm from a 21″ LED monitor. They placed their heads on a fixed chinrest and viewed the monitor split into two halves by a centrally placed wooden screen. Participants were also asked to wear prism glasses to ensure that images projected onto each eye were parallel.

#### Procedure

Participants were first tested for visual acuity and underwent a quick check to ensure that the CFS setup merged two objects present binocularly. After this, the main experiment began. All experiments reported here had trials that were self-initiated by a keypress. Participants were told that a colorful image of flashing circles would be presented on one side of the screen, and a picture of a face would be shown on another side of the screen. Participants were informed that they would see these images as overlapping in their view. They were told to expect to initially see only the flashing circles, and then slowly the image of the face would appear. They were asked to press the spacebar on the keyboard as soon as they saw the face. Participants were also told that on some trials, they would not be able to see a face because it may not break suppression or because there was no face on that trial at all. In such cases, they were asked not to press any key.

Each trial of the experiment began with an initial period of blank flickering. That is, for the 500 ms, only the suppressor was presented on either side of the screen. It flickered at one of four flicker frequencies (1, 4, 10, or 25 Hz). After 500 ms, a face was presented on the other side of the screen. The contrast of the face was ramped up in equal steps split over 1000 ms to avoid abrupt onset effects. The contrast of the target picture ramped up to 60% and stayed at the value. The mask continued to flicker along with the target on the other side until the participant pressed a key or a trial timed out (after 10 s). The target and mask locations were counterbalanced across trials. Each face was presented 14 times for each flicker frequency (14 × 4) on either side of the screen (14 × 4 × 2) making for a total of 112 trials. The breakthrough suppression duration was averaged across different flicker frequency trials (28 trials for each) for only target-present trials. Additionally, 15% of the trials were added as catch trials. In the catch trials, no face appeared at all during a trial. Participants were instructed not to press the response key if they did not see a face. These catch trials ensured participants followed instructions and did not just press the response key arbitrarily. If a participant had more than a 20% error rate in catch trials, they were to be excluded. No participant failed this exclusion criterion.

### Results Experiment 1

In the results section of all the experiments, we first report results from analysis of variance (ANOVA) and then *post hoc t*-tests. The post-tests are corrected for multiple comparisons using the Holm method. The number of tests reported is the number of tests that were performed.

**Figure 3:**
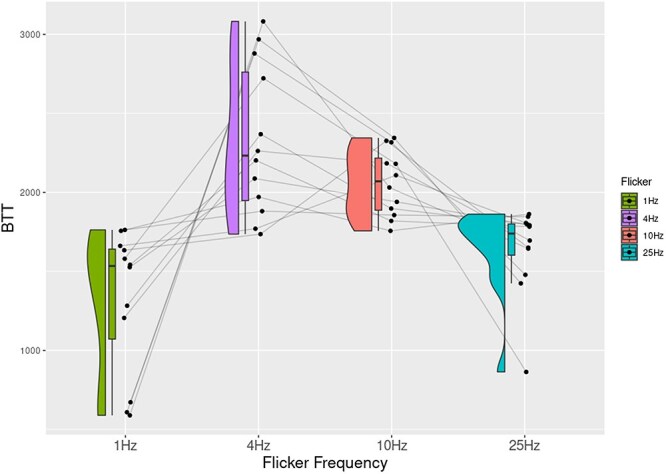
The plot shows normalized breakthrough times (BTT) across different flicker frequencies. Individual participants’ data and their trends are shown through connected lines.

For Experiment 1, we averaged breakthrough times for target-present trials separately for each of the four different flicker frequencies. Almost all participants performed at near perfect accuracy (only four participants missed a target, with a maximum miss rate of 2.7%). Participants also did not respond arbitrarily, only five participants reported seeing a face when there was none (false alarm), with very rare occurrences (maximum twice out of 16 catch trials). A one-way repeated measures ANOVA was performed on averaged breakthrough times with flicker frequency as the independent variable. Our results showed a significant main effect of flicker frequency on breakthrough times, *F*(3, 33) = 13.1, *P *< 0.001, η^2^ = 0.54. *Post hoc* tests revealed that participants reported that the target face broke into their awareness slowly when the mask flickered at 4 Hz (Fig. 3; Table 1).

**Table 1.** Values from corrected *t*-tests for Experiment 1.

4-Hz flicker compared	*t*-statistic	Holm-corrected *P*-value	Cohen’s *d*
1 Hz	5.75	<.001	1.07
10 Hz	2.06	.06	0.59
25 Hz	3.94	<.001	0.74

## Experiment 2

### Method

#### Participants

The same number of participants (*N* = 12) were again recruited for Experiment 2 (three females, mean age = 27.7 years).

#### Apparatus

The apparatus remained the same as in Experiment 1.

#### Procedure

Each trial began with a blank period of 500 ms where only the suppressor flickered on any one side of the screen. Over 1000 ms, an oval appeared on the other side of the screen. The contrast of the oval was ramped up in equal steps over this interval. This oval target was presented inside a square box. The oval appeared close to one of the four edges of the square. In each trial, the position of the oval was slightly jittered (∼1°) to avoid adaptation effects. Participants were asked to indicate by a keypress which edge the oval appeared closest to. If they did not respond within 10 s, the trial timed out. Since this was a discrimination task, we did not use catch trials. The experiment had a total of 144 trials (four locations × four flicker frequencies × two sides of the screen × four repeats).

### Results Experiment 2

**Figure 4:**
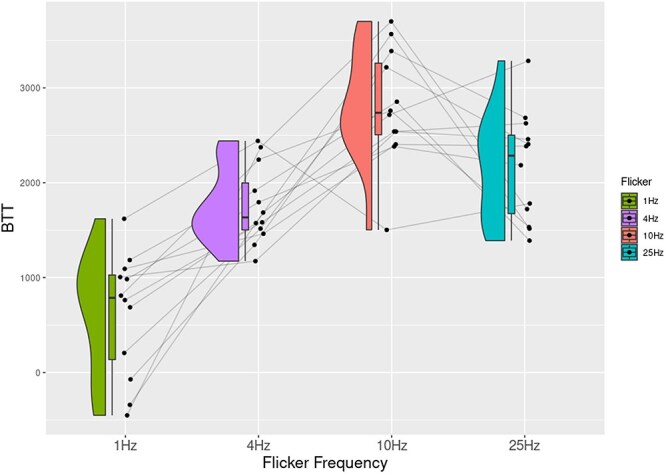
The plot shows the time it takes participants to find the target, as a function of flicker frequencies. Individual data points are shown.

To test the efficacy of the flicker frequencies of the suppressor, we averaged response times across participants separately for each flicker frequency. A repeated measures one-way ANOVA showed a main effect of flicker frequency, *F*(3, 33) = 23.2, *P *< .001, *η*^2^ = 0.68. *Post hoc* comparisons showed that participants were slowest to identify the location of the hidden oval target in trials with a 10-Hz flicker (Fig. 4; Table 2).

**Table 2.** Values from corrected *t*-tests for Experiment 2.

10-Hz flicker compared	*t*-statistic	Holm-corrected *P*-value	Cohen’s *d*
1 Hz	8.11	<.001	2.66
4 Hz	3.86	<.001	1.27
25 Hz	2.36	.048	0.77

## Experiment 3

### Method

#### Participants

We recruited the same number (12) of participants for the experiment (five females, mean age = 26.2 years) as in other experiments.

#### Apparatus and Stimuli

The apparatus remained the same as in other experiments. We used four Pacman-like figures that were arranged to form an illusory Kanizsa square or the same arranged in an arbitrary fashion where they did not form any shape (rotated 180°). The stimulus set encompassed a square region approximately subtending 3° × 3° visual angle.

#### Procedure

This experiment was also a bCFS task similar to Experiment 1. Participants were asked to press a key as soon as a target broke through into their awareness. They had to report only target breakthrough and not target identity. The instructions did not require participants to discriminate between different stimulus organizations. After a blank period of 500 ms, the image of the geometric set ramped up in equal steps over 1000 ms. The image remained on the screen for 10 s, while the suppressor continued flickering. A trial ended when participants reported a breakthrough of the target. Otherwise, in the absence of a keypress, the trial timed out after 10 s. Catch trials were randomly placed (16 trials) where no image was displayed, and these acted as tests for false alarms and arbitrary responding.

### Results Experiment 3

**Figure 5:**
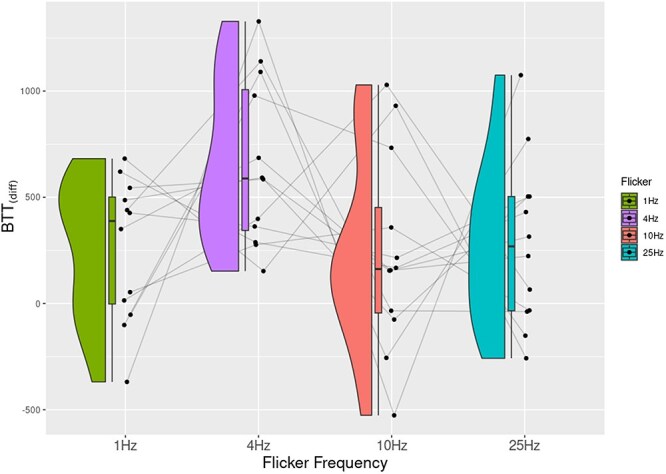
The plot shows the difference in breakthrough times between trials with and without an illusory contour. The benefit for illusory targets breaking out faster is only present for trials with low suppressor flickers.

Breakthrough times were averaged across stimulus configurations (Kanizsa and non-Kanizsa) and flicker frequencies, and a two-way repeated measures ANOVA was performed. (Normality assumption was violated for two levels of the data; however, ANOVA results are still reported for consistency with analyses from other experiments. Nonparametric alternates of ANOVA show the same significant effects.) The results showed a main effect of both flicker frequency, *F*(3, 33) = 21.6, *P *< .001, *η*^2^ = 0.59, and stimulus configuration, *F*(1, 11) = 5.1, *P =* .039, *η*^2^ = 0.03. Our results showed that overall, stimulus configurations that gave rise to an illusory Kanizsa square broke out faster than the stimulus configuration that did not. Since we were interested in seeing whether this was true for all flicker frequencies, we did pairwise comparisons for each. This comparison was done separately for each flicker frequency. Our expectation was that breakthrough benefit for the illusory contour would reduce as the flicker frequency increased. Our results were consistent with this expectation (Fig. 5; Table 3). First, a significant breakthrough benefit for the illusory Kanizsa square was present only when the mask flickered at 1 and 4 Hz, and there were no significant differences for flicker frequencies of 10 and 25 Hz . Moreover, the effect size for these differences reduced with increasing flicker frequency, with the smallest effect size for the fastest flickering trials (Table 3).

**Table 3.** Values from corrected Wilcoxon tests for Experiment 3.

Flicker frequency (Hz)	*Z*-statistic	Holm-corrected *P*-value	Effect size (biserial correlation)
1	2.51	.009	0.82
4	2.35	.016	0.77
10	1.17	.26	0.39
25	0.78	.47	0.26

We also sought support in favor of the null hypothesis that trials in which the mask flickered at 10 and 25 Hz offered no advantage to the illusory Kanizsa square. This was done to specifically look for evidence supporting a null effect for these flicker frequencies. A Bayesian paired *t*-test showed that there was weak evidence to support this null hypothesis (Bayes’ factor in favor of null = 2.8 and 2.7, respectively). These values are close to the cut-off for moderate evidence (Bayes’ factor of 3) in favor of the null hypothesis.

Since Experiment 3 was also a breakthrough task like Experiment 1, one would expect that overall breakthrough times would be slowest for trials with a suppressor flicker frequency of 4 Hz. On averaging trials across the stimulus configurations, we found that breakthrough was indeed slowest at 4 Hz, this replicates the results from Experiment 1 for non-face stimulus sets as well (see the Supplementary material for results).

## Experiment 4

### Method

#### Participants

Another pool of 12 participants (mean age = 25.8; four females) were recruited for the experiment.

#### Apparatus and stimuli

The apparatus remained the same as in other experiments. The pictures of the same male and female faces from Experiment 1 were used.

#### Procedure

This experiment was a modification to Experiment 1. Instead of reporting breakthroughs, participants were asked to press a key as soon as a target broke away from their awareness. A face was presented on the screen that was completely visible to the participant and over time would be suppressed by the flickering circles on the other side of the screen. The face was presented from the beginning at 60% contrast for 2 s and then was ramped down in contrast (reduction in 10% contrast every second). Participants were asked to press a key as soon as the face seemed to disappear behind the suppressor. The trial timed out after a total duration of 10 s. To ensure that participants were not randomly responding, 16 catch trials were used where the face never ramped down in contrast and stayed on the screen, and these trials were shorter in duration (5 s). The catch trials were presented for a shorter duration since the face would not get suppressed so soon with any reduction in contrast and this would ensure that these trials would act as a valid “catch” for arbitrary responding. Along with 96 main trials (24 trials per flicker frequency), this added up to 112 trials.

### Results Experiment 4

**Figure 6:**
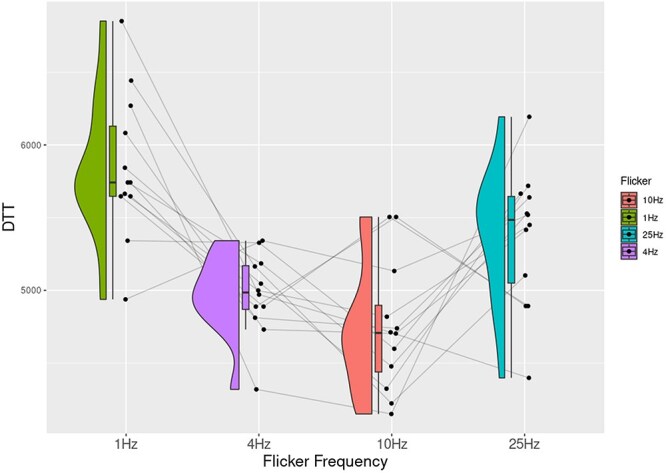
The time it takes for the target to disappear (DTT) from awareness is plotted as a function of flicker frequencies, showing the longest times for trials with a flicker of 1 Hz.

Here, we compared the disappearance times across different flicker frequencies using a one-way repeated measures ANOVA. The results showed a main effect of flicker frequency, (*F*(3, 33) = 11.18, *P *< .001, *η*^2^ = 0.50). Our results showed that targets took longest at a flicker frequency of 1 Hz, compared to the others (Fig. 6; Table 4).

**Table 4.** Values from corrected *t*-tests for Experiment 4.

1-Hz flicker compared	*t*-statistic	Holm-corrected *P*-value	Cohen’s *d*
4 Hz	4.26	<.001	0.99
10 Hz	5.39	<.001	1.26
25 Hz	3.94	.07	0.55

## Discussion

In this study, we demonstrate that different aspects of our visual experience evolve and devolve at different timescales by linking different flicker frequencies in CFS to perturbations in different visual tasks. In Experiment 1, we showed that visual contents broke slowly into awareness in trials when the CFS flickered at 4 Hz compared to the other three flicker frequencies. Similarly, in Experiment 2, a flicker of 10 Hz maximally perturbed the search for the spatial location of a target. Orthogonally, Experiment 3 showed that breakthrough benefits of perceptual organization of illusory contours were reduced most at faster-flicker frequencies of 25 Hz. Finally, we also investigated the timescale of content devolving away from awareness in a reverse CFS task where participants reported disappearance of targets. Contents disappeared slowly when the CFS mask flickered at the slowest frequency (1 Hz).

### Evidence for multiple timescales of experience

In general, theories of consciousness limit evolution and devolution of contents to single timescales (for a critical review, see Kent and Wittmann 2021). We had derived the values for temporal extension (300–500 ms) of the intermediate level of the framework from empirical data investigating time taken for a stimulus to persist in conscious awareness (Atmanspacher et al. 2008; Dainton, 2010) and upper limits of integration cycles within conscious experiences (Herzog et al. 2020). To maximally inhibit the temporal regularity of extensional aspects of our experience, we hypothesized in Experiment 1 that a face would slowly breakthrough in participants’ awareness at a flicker frequency of 4 Hz. Results from Experiment 1 supported this prediction. Not only did the range of flicker frequency match the purported oscillatory mechanisms in updating of extended visual contents (theta range, Doesburg et al. 2009), but also the estimates from our hierarchical framework. Our study is not the only study to show that breakthrough times of images are largest in this range (Zhu et al. 2016).

We used the standard flicker rate used in CFS of 10 Hz to have a bench-marked comparison across tasks and flicker frequencies and to identify a task, which maximally perturbed at the standard flicker rate. Experiment 2 results showed that this was the case when participants had to locate a dim target in one of four possible locations and were slowest to perform this task with a 10-Hz flicker. A reason for this could be the rhythmic attentional sampling in this frequency range (Landau and Fries 2012). It is to be noted that the target also shared feature similarity with the flickering CFS mask (the target being oval and the mask being made up of circles).

Similarly, in our framework, we had assigned a cinematic-like phenomenological mode to the fast-updating level with a temporal regularity between 30 and 50 ms. In Experiment 3 of this study, we showed that when this level is disrupted with a flicker frequency within this range (25 Hz), the stimulus advantage of illusory contours breaking through faster is lost. Another study done by Kaunitz et al. (2014) showed that brighter targets broke out faster and this effect reduced and disappeared when the flicker frequency was increased to 28.5 Hz. Given that previous studies which have shown that illusorily grouped contours break out faster from suppression in CFS (Wang et al. 2012) because the illusory contour has a higher perceived brightness, these two studies are compatible with our results. Like contrast effects disappearing at fast flickering frequencies (Kaunitz et al. 2014), it stands to reason that an early perceptual grouping effect, which leads to perceiving an illusory surface as brighter than a background, would also lose its advantage in the same flicker frequency range. Our work here helps to reconcile the results across these three findings. The results in Experiment 3 show no advantage of illusory contours at 10 or 25 Hz. Even though we only predicted a null for a flicker frequency of 25 Hz, it is unclear whether the lack of an effect at 10 Hz is interpretable. There is a parallel line of research, which argues that the priority in perceiving shapes with illusory contours is modulated by allocation of spatial attention (Wittenhagen and Mattingley 2019), this could account for null results even in trials with a flicker frequency of 10 Hz. However, this issue needs further empirical investigation to pin down the role of spatial attention and illusorily brighter contrast in breakthrough benefits noted under CFS.

In Experiment 4, we modified the CFS paradigm for a novel investigation into how a seen target content would devolve out of visual awareness as a function of flicker frequency. Estimates of specious presents (it is an idea in phenomenology where experience is thought to have an extended temporal window: the length of the window is that which is sufficient to maintain the persistence of just-past experienced contents while experiencing new ones, for instance, listening to a melody as a whole, instead of single notes; Dainton 2010) and psychological “nows” have been in the range of a few seconds, where it is argued that this is the duration over which experienced content is immediately accessible before fading away or being encoded into memory (Dainton 2010). First, all breakthrough times across experiments fall roughly within the estimated ranges of the specious present (∼3–5 s). These results are comparable to dwell times in binocular rivalry and bistable perception (Pöppel 1997). Given that this was a reciprocal CFS task, we expected that the frequency closest in temporal regularity to the retentional level would delay the exit of the face from awareness. This is what we saw in the results of Experiment 4. A possible reason for this is that the slower flicker frequency may act as an entraining anchor for the visual content over time.

### Preconscious or concurrent perturbations of experience

There are two ways in which our results can be interpreted based on the conceptualization of how experience unfolds. For instance, it could be the case that experience occurs at the end of a buffer period after sufficient nonconscious processing has occurred. Within time consciousness and phenomenology, these views are formulated under cinematic and dynamic snapshot models [see Dainton (2010) for a review]. Here, awareness is delayed and has no temporal extent. In this view, our multi-timescale manipulations would perturb processes unfolding within this preconscious buffer period, and its effects would be apparent after the buffer period had ended (Prosser 2017). In an alternative formalization that we prefer, visual experience could be construed as being extended over time, with its evolution being constrained by processes occurring faster or slower than its unfolding. Models of such kind propose a hierarchical structure to experience, where contents of experiences are constrained across multiple timescales as they unfold (Kent and Wittmann 2021, Singhal and Srinivasan 2021). These two views favor different representational formats and argue for remarkably different dynamics of experience [see Kiverstein and Arstila (2013) for a review]. In this study, we do not resolve this debate; however, for empirical work on these distinctions, we refer the reader elsewhere (Moutoussis and Zeki 1997, Singhal and Srinivasan 2024).

### Recommendations and reconciling flicker frequency choices in CFS experiments

While we also argue that the generic use of 10-Hz flicker frequency is not optimal (Zhu et al. 2016, Drewes et al. 2018; Han et al. 2018), our suggestions differ from previous studies. We show that the choice of flicker frequency depends on the phenomenal nature of the paradigm being employed. For instance, when using bCFS designs, our recommendation is to use flicker frequencies close to 4 Hz, since it best tracks the temporal regularity of contents breaking into awareness. This is based on the temporal regularity of contents breaking into awareness being in the range of 4 Hz. When the suppressor discretely flashes at this rate (4 Hz), it competes with the target in entering awareness leading to longer breakthrough times.

If investigators are interested in eliminating prioritized access to targets when the perceptual aspect of interest is related to early visual organization (grouping, contrast effects, brightness, and so on), faster flicker frequencies in the range of 25 Hz may work best. This is because early visual processes have been shown to unfold relatively fast within these timescales, the suppressor when flickering at similar timescales perturbs perceptual activity relevant to this early visual processing. Along the same lines, a flicker of 10 Hz (which is the standard in CFS tasks) possibly perturbs attention-related processes that operate within the alpha band rhythms (Landau and Fries 2012).

Finally, very slow flickering Mondrians can be used to delay the decay and devolution of content from visual awareness. Han et al. (2016) showed that when suppressors in a CFS task are updated smoothly over time (instead of discrete updates), very low flicker frequencies show peak suppression (0.3–1.8 Hz). We offer a possible reconciliation of these results with Experiment 4 of our study, which shows that when the target is visible from the beginning it takes the longest for it to be suppressed at the slowest flicker frequency (1 Hz). One could argue that this is simply because 1 Hz does not adequately suppress targets. However, this is not the case with results of Han et al. (2016). It is our speculation that when the suppressor changes smoothly (unlike discrete flashes used here and elsewhere), it does not compete for entry into awareness but continues to be “retained” in awareness. Thus, when the mask flickers smoothly at 1 Hz, the mask itself takes longer to decay from awareness, resulting in a longer duration for the target breaking through into awareness. This contrasts with discretely flashing masks, which delay target breakthroughs into awareness at a distinct temporal regularity (1 vs. 4 Hz). Han et al. (2016) argue that binocular rivalry and CFS may result from the same underlying mechanisms since their suppression dynamics are similar. Here, we argue that CFS perturbs distinct visual processes depending on the flicker rate of the suppressor, and thus CFS and binocular rivalry may reflect different temporal aspects of a similar perceptual process.

Another reason why Han et al. (2016) find similarities between CFS and binocular rivalry is possibly due to the nature of flickering of the suppressor in their study. Han et al. (2016) used a continuously changing suppressor. Such a manipulation may present itself in experience as a persisting visual object undergoing changes, making it similar to binocular rivalry where two distinct objects are presented binocularly. In contrast, using a flash-based strategy for the suppressor that refreshes the suppressor object in every flicker resets the experience of the suppressor at each moment. We speculate that this phenomenological difference of suppressor persistence vs. resetting may account for differences between our results and Han et al.’s (2016) results and also hint at possible differences between CFS and binocular rivalry in general.

In this study, we used the nested hierarchical framework of time, which we previously proposed, to predict which flicker frequency would maximally inhibit specific aspects of visual experience. This helps reconcile different results of temporal frequency investigations under CFS, with studies showing peak suppression at remarkably different flicker frequency bands [see Han et al. (2016), Zhu et al. (2016), Tsuchiya and Koch (2004, 2005), and Kaunitz et al. (2014): 1, 4, 10, and 25 Hz, respectively]. Additionally, we also account for the phenomenological distinctions across these disparities of flicker frequencies. For instance, we account for why breakthrough tasks are suppressed at different rates based on the change of the suppressor, smoothly or in discrete flashes (1 and 4 Hz). This is possible because the smooth suppressor perturbs a slower retentional aspect of visual awareness, while the discrete suppressor competes with the target for entry into awareness. Similarly, fast flickering suppressors (25 Hz) perturb early visual processes that constrain the contents of awareness, maximally suppressing brief targets and eliminating breakthrough priority based on perceptual differences (like in Experiment 3). Here, we demonstrate how these disparate results can be reconciled under an understanding of multi-timescale evolution of experience. Specifically, the entry of contents into awareness, spatial attentional sampling, early visual grouping, and decay of contents of awareness all occur at distinct timescales. These results support the growing view of multi-timescale visual awareness, which argues that experiences evolve and devolve over distinct temporal regularities (Aru and Bachmann 2017, Singhal and Srinivasan 2021).

The present work did not investigate the neural mechanisms responsible for this perturbation. However, follow-up experiments could try to understand the basis for these perturbations under an oscillatory framework where perturbations brought about the flickering of the suppressor hamper communications across different frequency bands (Jirsa and Müller 2013). Future studies can also use the same framework as a tool for picking flicker frequencies for various CFS tasks.

### Limitations

While our study attempts to demonstrate a multiple-timescale view of visual awareness and advance theories of consciousness, the study has some limitations. Our study used only four flicker frequencies to represent different bands and regularities of experience. While we report unique perturbations for each of these, a study with greater granularity of frequency ranges is required to be more precise about the impact of flicker frequencies on visual experience. To further strengthen the link between timescales of visual awareness and perturbations via CFS flicker frequencies, a future study could also look to concurrently measure both neural activities (e.g. brain oscillations) and behavioral responses.

We also used different stimuli for three of the four different experiments. Even though we feel it was necessary (target-distractor similarity in the case of Experiment 2 and illusory contours in the case of Experiment 3), it is possible that this could have led to low-level confounds. For example, our results could have been confounded by spatial frequencies or complexities of the stimulus. Further detailed studies are required to rule out these confounds. A design that would use similar stimuli but manipulate the task or a design that would have different stimuli controlled for low-level differences but use the same task may be required to conclusively demonstrate the effect of different flicker frequencies.

Previously, it has been argued that CFS suppressors inadvertently possess high power at very low frequencies (Han et al. 2016). To avoid this confound, we used a tool prescribed by the same research group for accurate control of flicker frequencies in CFS (Wang et al. 2023). Our results demonstrate that distinct flicker rates perturb different properties of visual awareness when flicker frequencies are controlled for precisely. However, our study used a flash-based strategy for flickering the CFS suppressor, in contrast with Han et al. (2016) who used a continuously changing suppressor. It is possible that this discrete vs. continuous manner of flashing suppressors in a CFS paradigm has unique temporal phenomenologies. For instance, continuously updating suppressors would have visual features persisting in experience as the mask continues to change, whereas in discrete flashes the experience of the mask resets in each moment of change. If the experience of a continuously updating mask is that of a persisting object undergoing changes—rather than new objects flashing in each moment—then for a range of tasks, peak suppression may only occur at low frequencies. This is because the suppressor would slowly exit awareness at frequencies that match anchors of experience (as seen in Experiment 4 for targets). Further studies are needed to compare and contrast these results both behaviorally and phenomenologically. It should also be noted that if the results in CFS are predominantly driven by low frequencies, then there should not be differences in results across different experiments, which we have demonstrated.

## Conclusion

In this study, we set out to offer support to the view that awareness evolves and devolves at distinct timescales. We modified a common paradigm used in consciousness research to create a new variant of CFS. Using the logic of a double dissociation, we showed that four different flickering frequencies inhibited performances in four different tasks of visual awareness. Flicker frequencies of 4 Hz maximally perturbed content breaking into awareness. Performance in sampling the location of a dimly presented target was slowed down by CFS masks flickering at 10 Hz. We demonstrated that the benefit of perceptually grouped illusory contours breaking into awareness disappears at fast-flicker rates (25 Hz). Finally, we showed that a slow flicker of the CFS mask at 1 Hz maximally delayed the devolution of visual contents from awareness. Together, the four experiments show how visual contents evolve and devolve out of our awareness simultaneously over multiple timescales.

## Supplementary Material

niaf005_Supp

## Data Availability

Data summaries are available for access in the online repository via the link: https://osf.io/d4m6z/
