# Supplementary material for: Deciphering temporal scales of visual awareness: insights from flicker frequency modulation in continuous flash suppression
Source: Neurosci Conscious. 2025 Mar 6;2025(1):niaf005. doi: 10.1093/nc/niaf005 (PMC11884739; doi:10.1093/nc/niaf005)
Supplement: Supplementary file 1 [file niaf005_supp.zip › suppl_data/SM_CFS.odt]

Supplementary Material

1. Apparatus Details

Spectacles: dioptre 10, base out

Wooden screen: Custom made. Adjustable for length from 90 to 130 cm.

Chinrest: Custom made metal chinrest. Adjustable such that participants head is vertically alligned with the wooden screen and they can comfortably view the monitor.

2. Flicker frequency comparisons for experiment 3

| 4Hz flicker compared | t statistic | Holm corrected p value | Cohen’s d |
| --- | --- | --- | --- |
| 1Hz | 8.02 | <0.001 | 1.5 |
| 10Hz | 3.72 | 0.002 | 0.69 |
| 25Hz | 3.35 | 0.004 | 0.63 |
